# The Relationships Between Self-Compassion, Attachment and Interpersonal Problems in Clinical Patients with Mixed Anxiety and Depression and Emotional Distress

**DOI:** 10.1007/s12671-017-0835-6

**Published:** 2017-11-06

**Authors:** Kate Mackintosh, Kevin Power, Matthias Schwannauer, Stella W. Y. Chan

**Affiliations:** 1 NHS Tayside Psychological Therapies Service, Tayside, Scotland UK; 2 Section of Clinical Psychology, School of Health in Social Science, University of Edinburgh, Edinburgh, EH8 9AG UK; 3 School of Natural Sciences, University of Stirling, Stirling, Scotland UK

**Keywords:** Self-compassion, Attachment, Interpersonal problems, Anxiety, Depression

## Abstract

Self-compassion has been consistently linked to psychological well-being. The ability to be self-compassionate may be shaped by early attachment experiences and associated with interpersonal difficulties. However, evidence has yet to be extended to clinical populations. This study examined the role of self-compassion and its relationship with attachment and interpersonal problems in clinical patients with anxiety and depression. Participants (*N* = 74; 60% female, mean age 40 years) were recruited from a primary care psychological therapies service in Scotland, UK. Participants completed four self-report questionnaires assessing self-compassion, attachment, interpersonal problems and emotional distress (including depression and anxiety). Low self-compassion, attachment-related avoidance (but not attachment-related anxiety) and high interpersonal problems were all associated with higher levels of emotional distress and anxiety. Low self-compassion and high interpersonal problems were predicted by attachment-related avoidance. Self-compassion mediated the relationship between attachment-related avoidance and emotional distress and anxiety. This was a cross-sectional design and therefore a definitive conclusion cannot be drawn regarding causal relationships between these variables. Self-reported questionnaires were subject to response bias. This study has extended the evidence base regarding the role of self-compassion in patients with clinical levels of depression and anxiety. Notably, our findings indicated that self-compassion may be a particularly important construct, both theoretically and clinically, in understanding psychological distress amongst those with higher levels of attachment avoidance. This study supports the development and practice of psychotherapeutic approaches, such as compassion-focused therapy for which there is a growing evidence base.

**Electronic supplementary material:**

The online version of this article (10.1007/s12671-017-0835-6) contains supplementary material, which is available to authorized users.

## Introduction

In the UK, one in four adults experience mental health problems in any one year, with anxiety and depression being the most common mental health problems (Singleton et al. 2003). There has been a growing research focus on identifying mechanisms that may protect individuals from developing mental health problems. One such mechanism is self-compassion. Neff (2003) conceptualised self-compassion as consisting of three components: self-kindness, common humanity and mindful acceptance. Self-kindness involves being emotionally warm and non-judgemental towards the self in times of difficulty; common humanity relates to recognising that life’s difficulties are part of human experience; and mindful acceptance refers to being able to acknowledge and observe painful thoughts and feelings (Neff 2003).

A recent meta-analysis indicated a large effect size for the association between higher levels of self-compassion and lower levels of psychopathology (MacBeth and Gumley 2012). However, a majority of the studies included in the meta-analysis were conducted with convenience samples of college students or community subclinical samples: of the 20 included samples, only one was recruited from clinical populations, involving adults with a diagnosis of Generalised Anxiety Disorder (Roemer et al. 2009). Since then, a handful of studies have been conducted reporting lower levels of self-compassion in individuals with depression (Krieger et al. 2013) and social anxiety (Werner et al. 2012) compared with healthy volunteers. Similarly, van Dam et al. (2011) found that self-compassion predicts symptom severity in a community sample seeking treatment for depressive and anxiety symptoms. Exploring self-compassion in clinical populations is of particular importance, given that treatment adherence and access to mental health facilities could be compromised by self-stigmatisation and subsequent reduction in self-esteem and personal empowerment (Corrigan and Calabrese 2003). Self-compassion may therefore potentially play a role in enhancing mental health outcomes through altering these negative mediators.

Whilst there is an emerging evidence base supporting the link between self-compassion and anxiety and depression, less is known about the origins of self-compassion. Both Gilbert (2005) and Neff and McGehee (2010) have linked the development of self-compassion with early attachment experiences. Gilbert (2005, 2009) proposed that self-compassion is the ability to soothe the self with kindness and non-judgemental understanding when presented with a threat or negative affect. His proposed model of compassion is theoretically linked to three interacting emotion regulation systems: threat, soothing and incentive seeking (Depue and Morrone-Strupinsky 2005). Gilbert (2010) hypothesised that negative attachment experiences may result in an overdeveloped threat system and an underactivated soothing system, therefore potentially leaving the child struggling to feel safe on their own and/or with others, leading to reduced ability to be compassionate. Similarly, Gillath et al. (2005) also suggested that the ability to self-soothe develops through being comforted by attachment figures in early life. Therefore, if this comfort is missing or inconsistent, the ability to self-soothe may not fully develop.

Consistent with the theory that compassion is rooted in early attachment experiences, self-compassion has been associated with maternal warmth and family functioning (Neff and McGehee 2010; Pepping et al. 2014), as well as childhood emotional and physical abuse, neglect and maltreatment (Tanaka et al. 2011; Vettese et al. 2011). Furthermore, adolescents with a secure attachment style reported greater self-compassion, whilst those with preoccupied or fearful attachment styles demonstrated lower levels of self-compassion (Neff and McGehee 2010). Low self-compassion was found to be significantly correlated with anxious and avoidant attachment (Pepping et al. 2014; Raque-Bogdan et al. 2011) and mediate the relationship between attachment and mental health outcomes (Neff and McGehee 2010; Raque-Bogdan et al. 2011; Wei et al. 2011). Finally, insecure attachment is associated with a fear of compassion from others (Gilbert et al. 2014). These findings further suggest that enhancing self-compassion can potentially be an important therapeutic target.

However, apart from Gilbert et al. (2014), all of the above studies have all involved non-clinical populations. Therefore, to be clinically useful, it is important to examine whether the link between self-compassion and attachment, along with the mediating role of self-compassion between attachment and psychopathology, can be replicated in the clinical population. Given that attachment has been strongly associated with psychopathology (Mikulincer and Shaver 2012), in particular depression and anxiety (Bosmans et al. 2010; Catanzaro and Wei 2010), examining the factors that mediate this relationship is of high theoretical and clinical value.

Furthermore, in Neff’s (2003) conceptualisation of self-compassion, one of the three core components is the interpersonal component of common humanity. However, the interpersonal aspect of self-compassion has not been widely explored. Neff and Beretvas (2013) suggested that being self-compassionate is associated with positive romantic relationships. Additionally, Yarnell and Neff (2013) found that when resolving romantic relationship conflicts, those with higher levels of self-compassion were more likely to compromise and balance the need of self and other, and less likely to experience emotional turmoil. Therefore, self-compassionate individuals may engage in more adaptive social interactions and relationships and have more adaptive reactions to difficult interpersonal situations, which may serve as protective factors against psychopathology.

Indeed, interpersonal problems have been associated with psychopathology (Girard et al. 2017). Although it has been proposed that depressed individuals may have a tendency to generate interpersonal stress (Coyne 1976), there has also been evidence indicating that interpersonal problems persist after recovery (Coryell et al. 1993), contribute to the development of depressive symptoms (Hammen et al. 2004) and mediate outcomes of psychotherapies (Bernecker 2012; Dinger et al. 2013). These are in line with the view that the link between interpersonal problems and psychopathology is likely to be bidirectional (Hammen 2006; Joiner 2002). Interestingly, in a 2-year longitudinal study on a sample of college students, Hankin et al. (2005) found that experiencing additional interpersonal stressors over time mediates the relationship between attachment insecurity and prospective increase in depressive and anxious symptoms. Given that interpersonal problems have been demonstrated to be linked to both insecure attachment and psychopathology (Mikulincer and Shaver 2005), it is important to explore these relationships further, especially in clinical populations.

Taken together, there is a growing body of literature indicating the link between self-compassion and psychological well-being, thereby supporting the development of therapies that enhance self-compassion. It has been proposed that the ability to be compassionate towards the self may be shaped by early attachment experiences, and there is some initial evidence that self-compassion mediates the relationship between attachment and anxiety and depression. However, to date, there has been no research exploring the relationships of these constructs in a clinical population. Additionally, interpersonal functioning is linked to both attachment and psychopathology, and in theory will be related to self-compassion. However, this hypothesis has yet to be tested empirically. This study aimed to explore the relationships between attachment, self-compassion, interpersonal problems and mental health in a clinical population with the following specific hypotheses: Firstly, lower levels of self-compassion and higher levels of interpersonal problems will be associated with attachment-related anxiety and/or avoidance. Secondly, lower levels of self-compassion will be associated with higher levels of interpersonal problems. Thirdly, self-compassion and interpersonal problems will mediate the relationship between attachment style and symptoms of anxiety and depression.

## Method

### Participants

Participants were adults aged 18 upwards, presenting with anxiety and/or depressive disorders to a primary care psychological therapies service in NHS Tayside. They were required to have a command of English to the extent necessary to complete the questionnaires and had to be able to give informed consent. Participants were excluded if they had a diagnosis of an Axis II disorder, psychosis, current substance misuse or high levels of suicidality.

A total of 134 individuals were approached and consented to take part in the study. Of these, 74 returned the completed questionnaire packs, indicating a 55% completion rate. See Table 1 for demographic details.

**Table 1:** Demographic characteristics of sample (*N* = 74)

Demographic variable	*N* = 74
Age in years	
Mean (SD)	40.3 (12.0)
Range	18–64
Missing	2
	*N* (%)
Gender	
Female	44 (59.5%)
Male	26 (35.1%)
Missing	4 (5.4%)
Ethnicity	
White British	69 (93.2%)
White other	3 (4.1%)
Asian British	1 (1.4%)
Missing	1 (1.4%)
Relationship status	
Married	24 (32.5%)
In a relationship	21 (28.4%)
Divorced	4 (5.4%)
Widowed	4 (5.4%)
Separated	2 (2.7%)
Single	17 (23%)
Other	1 (1.4%)
Missing	1 (1.4%)
Employment status	
Employed	48 (64.9%)
Unemployed	9 (12.2%)
Student	9 (12.2%)
Retired	1 (1.4%)
Unable to work	6 (8.1%)
Missing	1 (1.4%)

### Procedure

This study employed a cross-sectional design. Ethical approval was granted by the East of Scotland Research Ethics Committee and Tayside Medical Science Centre Research and Development Office. Patients attending the Psychological Therapies Service who met the inclusion criteria were initially given the participant information sheet by their clinician. Those who expressed an interest in participating in the study were asked to complete the consent form, and then were provided with the study pack containing the questionnaires to take home and complete. They were asked to return the questionnaires to the researcher either via post or by handing the pack back to their clinician in a sealed envelope.

### Measures

#### The Self-Compassion Scale

The Self-Compassion Scale (SCS; Neff 2003) is a 26-item questionnaire measuring levels of self-compassion. Each item is rated on a 5-point Likert scale, ranging from 1 (almost never) to 5 (almost always). A higher overall mean score indicates a higher level of self-compassion. High internal consistency has been reported with Cronbach’s alpha reported as .92 (Neff 2003), .91 (Krieger et al. 2013) and .96 (Werner et al. 2012). In the current study, Cronbach’s alpha for the full scale was .71.

#### Hospital Anxiety and Depression Scale

The Hospital Anxiety and Depression Scale (HADS; Zigmond and Snaith 1983) consists of 14 items split equally into two subscales: anxiety and depression. The scoring range for each subscale is 0–21. A total score, indicating overall emotional distress, can also be calculated by summing all the items (Crawford et al. 2001). Interpretations of the scores for both subscales are based on the following cut-offs: 8–10, mild symptoms; 11–15, moderate symptoms; 16 or above, severe symptoms (Snaith and Zigmond 1994). A literature review of 747 studies concluded that the HADS demonstrated good concurrent validity, internal reliability and discriminant validity (Bjelland et al. 2002). In the current study, Cronbach’s alphas for the full scale, anxiety subscale and depression subscale were .87, .78 and .82, respectively.

#### Inventory of Interpersonal Problems 32

The Inventory of Interpersonal Problems 32 (IIP-32; Barkham et al. 1996) is a 32-item self-report measure assessing difficulties in interpersonal relationships. Items are scored on a 5-point scale ranging from 0 (not at all) to 4 (extremely). In a clinical sample of participants, McEvoy et al. (2014) reported a Cronbach’s alpha of .88 for the total score. In the current study, Cronbach’s alpha was .82.

#### The Experiences in Close Relationships-Revised

The Experiences in Close Relationships-Revised (ECR-R; Fraley et al. 2000) is a self-report measure with 36 items measuring adult romantic attachment across two subscales: attachment-related anxiety (fear of abandonment and rejection) and attachment-related avoidance (fear of closeness and discomfort with dependence on others). Participants rate on a 7-point Likert scale (1–disagree strongly, 7–agree strongly) how accurately each item describes their experience of close relationships. Sibley et al. (2005) provided support for its short-term temporal stability, two-factor structure and convergent and discriminant validity. Good internal consistency was reported by Raque-Bogdan et al. (2011), with Cronbach’s alpha at .92 and .94 for the avoidance and anxiety subscales, respectively. In the current study, Cronbach’s alpha for the avoidance subscale scale was .78 and for the anxiety subscale was .91.

### Data Analyses

Power calculation was conducted a priori based on Cohen (1988). Given the variability of effect sizes between attachment and self-compassion (Raque-Bogdan et al. 2011; Wei et al. 2011), the current study assumed a medium effect size. In order to have 0.8 power to detect a medium effect size when carrying out correlation/multiple regression analysis with three independent variables, a sample size of 76 would be required. A similar target sample size was estimated using Green’s (1991) formula (*N* ≥ 50 + 8 m) for multiple regression. For mediation analysis using a bootstrapping approach, Fritz and Mackinnon (2007) recommended that in order to achieve a power of 0.8 to detect a medium effect size of the indirect effect, a sample size of 71 would be required. The current sample size was therefore deemed sufficiently powered.

Statistical analysis was conducted in IBM SPSS Statistics Version 21. Mediation analysis was conducted using the computational and modelling tool PROCESS v.2.15 (Hayes 2013). Pearson correlations were used initially to explore the associations between attachment, self-compassion, interpersonal problems and emotional distress. Multiple mediation analysis was then carried out to explore self-compassion and interpersonal problems as possible mediators in the relationship between adult attachment style and emotional distress. Mediation analysis was conducted using the bootstrapping resampling method using 5000 bootstrap resamples (Preacher and Hayes 2008). Bootstrapping is a non-parametric method that estimates the indirect effect and its 95% confidence intervals. When the bias-corrected confidence intervals (BC CIs) do not contain 0, it is assumed that the indirect effect is significantly different from 0 at *p* < 0.05, thereby suggesting that the effect of the independent variable on the dependent variable is mediated by the proposed mediating variables.

Box plots were used to examine any outliers that could bias the data set. To establish whether data was normally distributed, values of skewness and kurtosis were converted to *z*-scores (Field 2013). All *z*-scores were non-significant indicating that the data could be assumed to be normally distributed.

Linearity and homoscedasticity were investigated by plotting the standardised residuals against the standardised predicted values (Field 2013). The scatterplots showed no obvious pattern, indicating that the assumptions of linearity and homogeneity of variance were met.

Finally, data was assessed for multicollinearity. High correlations between independent variables (.80 or greater) may indicate collinearity between variables, which would suggest they may not be appropriate for inclusion in mediational analysis. Pearson correlations were all less than .80 suggesting no evidence of collinearity. The variance inflation factor (VIF) and tolerance statistics were also used to assess for collinearity in the data. All the VIF values were well below 10 and the tolerance statistics all above 0.2, with the average VIF 1.261.

Following Fox-Wasylyshyn and El-Masri (2005), if more than 10% of items were missing on any questionnaire, it was excluded from further analysis. This resulted in four participants’ ECR-R data and two participants’ IIP-32 data being excluded from the analysis involving these variables.

Sixteen questionnaires had ≤ 10% missing data (three SCS, two HADS, three ECR-R and eight IIP-32). Various methods for imputing missing data have been described in the literature (e.g. Fox-Wasylyshyn and El-Masri 2005; Roth et al. 1999; Shrive et al. 2006). The person mean substitution method was used in the current study: the individual’s mean for the relevant scale/subscale was used to replace the missing values.

## Results

Descriptive statistics are presented in Table 2. Mean scores for HADS anxiety and depression indicated moderate levels of anxiety and mild levels of depression within the sample. Paired samples *t* test indicated that participants reported significantly higher anxiety symptoms than depressive symptoms (*t*(73) = 10.61; *p* < 0.001). Independent samples *t* tests indicated there were no gender differences on the SCS, ECR-R or IIP-32.

**Table 2:** Descriptive statistics and bivariate correlations between attachment style, interpersonal problems, self-compassion and emotional distress (associated *p* values)

	*N*	Mean	SD	Correlations between variables					
				ECR-R (avoidance)	ECR-R (anxiety)	SCS	IIP-32	HADS total	HADS anxiety
ECR-R (avoidance)	70	3.60	1.18	–					
ECR-R (anxiety)	70	4.04	1.39	0.363** (0.002)	–				
SCS	73	2.21	.46	− 0.255* (0.033)	− 0.247* (0.040)	–			
IIP-32	72	58.89	15.33	0.363** (0.002)	0.444** (0.000)	− 0.192 (0.107)	–		
HADS total	74	21.2	6.86	0.314** (0.008)	0.208 (0.083)	− 0.277* (0.018)	0.361** (0.002)	–	
HADS anxiety	74	13.07	3.92	0.321* (0.007)	0.192 (0.112)	− 0.347** (0.003)	0.323** (0.006)	0.860** (0.000)	–
HADS depression	74	8.14	4.03	0.215 (0.073)	0.164 (0.175)	− 0.130 (0.274)	0.300* (0.010)	0.868** (0.000)	0.493** (0.000)

*p < 0.05; **p < 0.01

Analyses were conducted to assess whether any demographic variables were related to the dependent variables (DVs), and hence should be included as covariates in the mediation analysis. Pearson correlations indicated that age was significantly correlated with HADS total (*r* = 0.272 *p* = 0.021) and HADS depression (*r* = 0.385 *p* = 0.001), but not HADS anxiety (*r* = 0.085, ns). Independent samples *t* tests indicated there were no gender differences on the HADS. One-way ANOVAs indicated that there were no significant differences in scores on the HADS based on relationship status or employment status. Therefore, age was the only demographic variable to be controlled for in the mediation analyses involving HADS total and HADS depression as the DVs.

Pearson’s correlations were conducted to explore the relationships between attachment, self-compassion and interpersonal problems (see Table 2). As hypothesised, self-compassion was negatively correlated with attachment avoidance and attachment anxiety, both with small to medium effect sizes. Attachment avoidance and attachment anxiety were also positively correlated with interpersonal problems, with medium to large effect sizes. There was no significant relationship between self-compassion and interpersonal problems.

In order to be included in the mediation or regression analysis, predictor variables should show a strong correlation with the DVs. In the current study, the possible predictor variables were self-compassion, anxious attachment, avoidant attachment and interpersonal problems. The DVs were overall emotional distress (HADS total), anxiety symptoms and depressive symptoms.

Attachment-related avoidance had a significant positive correlation with overall emotional distress and anxiety symptoms, both of medium effect size. There was no significant correlation between avoidant attachment and depressive symptoms, or between anxious attachment and overall emotional distress, anxiety symptoms and depressive symptoms. See Table 2.

Self-compassion had a significant negative correlation with overall emotional distress and anxiety, with small to medium and medium effect sizes, respectively. Interpersonal problems had a significant positive correlation with overall emotional distress, anxiety symptoms and depressive symptoms, all of medium effect size. There was no significant relationship between self-compassion and depressive symptoms. See Table 2.

**Fig. 1:**
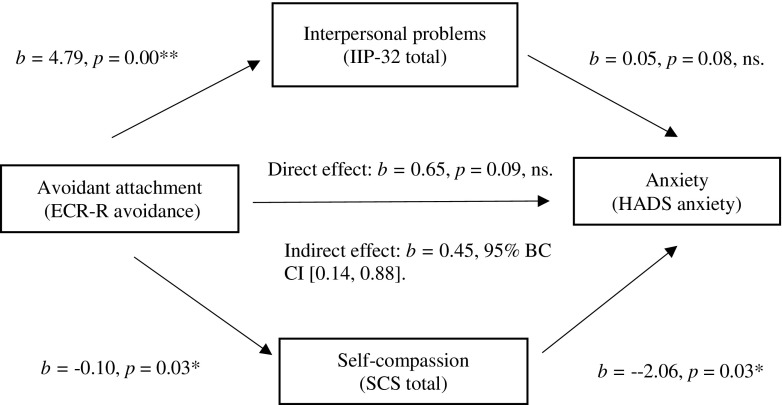
Mediation model—emotional distress as the dependent variable

It was hypothesised that self-compassion and interpersonal problems would mediate the relationship between attachment and emotional distress. This model is presented in Fig. 1. Multiple mediation analyses were conducted with attachment-related avoidance (ECR-R avoidance) as the independent variable (IV) and self-compassion (SCS) and interpersonal problems (IIP-32) as the mediators. Attachment-related anxiety (ECR-R anxiety) was not included in the mediation analysis due to a lack of relationship between this variable and the DV. As there is an overlap between anxiety and depressive symptoms, and in this study a significant correlation between the two subscales of the HADS, the total HADS score was used as a measure of overall emotional distress. Therefore, the main mediation analysis included emotional distress (HADS total) as the DV. Age was entered as a covariate.

To examine whether direct and indirect effects differed when treating anxiety and depression as separate constructs, four mediation analyses were further conducted, firstly with HADS anxiety as the DV. This model was then repeated with depression included as a covariate of anxiety. Although there was only a significant correlation between one of the predictor variables (interpersonal problems) and HADS depression, to allow comparisons with the anxiety model, the same mediation was conducted with HADS depression as the DV.

The results of the regression analysis are presented in Table 3. Both mediators were predicted by the IV: attachment avoidance significantly predicted self-compassion (*F*(1, 65) = 4.53, *p* = 0.04), and interpersonal problems (*F*(1, 65) = 11.63, *p* < 0.001). Attachment avoidance explained 7% of the variance in self-compassion and 15% of the variance in interpersonal problems.

**Table 3:** Results of regression analysis predicting self-compassion, interpersonal problems and emotional distress, controlling for age

Predictor variable	Outcome variable	Coefficient	SE	*t*	*p*
ECR-R (avoidance)	SCS	− 0.10	0.05	− 2.13	0.04*
ECR-R (avoidance)	IIP-32	5.16	1.51	3.41	0.00**
	Dependent variable				
	HADS total				
ECR-R (avoidance)		1.70	0.67	2.52	0.01*
SCS		−3.19	1.56	−2.05	0.04*
IIP-32		0.06	0.05	1.26	0.21

*p < 0.05; **p < 0.01

When the mediators were not included in the model, attachment avoidance significantly predicted emotional distress (*b* = 2.36, *t* = 3.81, *p* < 0.001) and accounted for 27% of the variance in emotional distress. When the three predictor variables were included in the model, they accounted for 34% of the variance in overall emotional distress. This model was significant (*F*(4, 62) = 7.80, *p* < 0.001). Of the individual predictors, self-compassion and avoidant attachment were significant when compared with the other predictors (*t* = − 2.05, *p* = 0.04 and *t* = 2.52, *p* = 0.01, respectively).

The total indirect effect of avoidant attachment on emotional distress through the two mediators had a coefficient of 0.65, with 95% BC CIs of − 0.0209 to 1.6395 (see Table 5 Supplementary Material). As the BC CIs cross 0, the total indirect effect is not significant. As the 95% BC CIs for self-compassion did not cross 0, although there was no total indirect effect, there was a significant indirect effect of avoidant attachment on emotional distress through self-compassion. Thus, there was partial support for the hypothesis that self-compassion and interpersonal problems would mediate the relationship between insecure attachment and emotional distress. This model is presented diagrammatically in Fig. 1.

Table 4 indicates attachment avoidance significantly predicted both self-compassion (*F*(1, 67) = 4.82, *p* = 0.03) and interpersonal problems (*F*(1, 67) = 10.18, *p* < 0.001). Attachment avoidance explained 6% of the variance in self-compassion and 13% of the variance in interpersonal problems.

**Table 4:** Results of regression analysis predicting self-compassion, interpersonal problems and anxiety

Predictor variable	Outcome variable	Coefficient	SE	*t*	*p*
ECR-R (avoidance)	SCS	− 0.10	0.05	− 2.20	0.03*
ECR-R (avoidance)	IIP-32	4.79	1.50	3.19	0.00**
	Dependent variable				
	HADS anxiety				
ECR-R (avoidance)		0.65	0.38	1.71	0.09
SCS		− 2.06	0.93	− 2.21	0.03*
IIP-32		0.05	0.03	1.75	0.09
Controlling for depression					
ECR-R (avoidance)		0.52	0.36	1.47	0.15
SCS		− 1.92	0.86	− 2.24	0.03*
IIP-32		0.03	0.03	0.98	0.33

*p < 0.05; **p < 0.01

When the mediators were not included in the model, attachment avoidance significantly predicted anxiety (*b* = 1.10, *t* = 3.00, *p* < 0.001). Without self-compassion and interpersonal problems in the model, attachment avoidance accounted for 11% of the variance in anxiety. When all three predictors were included in the model, they accounted for 22% of the variance in anxiety. This model was significant (*F*(3, 65) = 6.23, *p* = 0.001). Of the individual predictors, self-compassion was significant when compared with the other predictors (*t* = − 2.21, *p* = 0.03). Controlling for depression did not produce notable changes to this result (see Table 6 Supplementary Material).

**Fig. 2:**
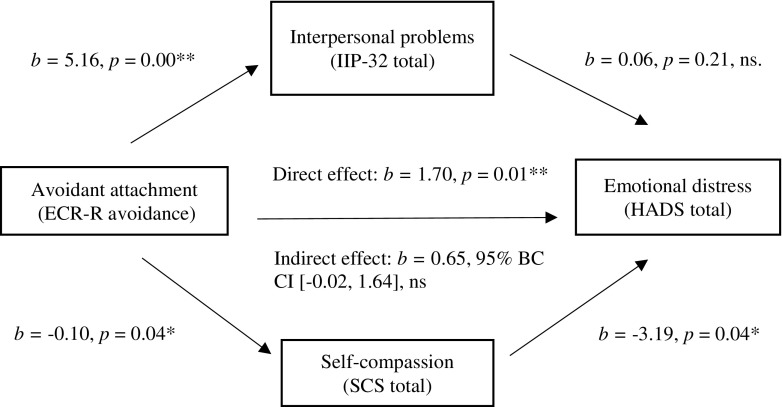
Mediation model—anxiety as the dependent variable

The total indirect effect of avoidant attachment on anxiety through the two mediators had a coefficient of 0.4463, with 95% BC CIs of 0.1400 to 0.8780. As these BC CIs do not cross 0, the total indirect effect is significant. Given the 95% BC CIs for both mediators did not cross 0, a significant indirect effect of avoidant attachment on anxiety through self-compassion and interpersonal problems was found. This is presented diagrammatically in Fig. 2. However, when depression was controlled for, interpersonal problems was no longer a significant mediator (see Table 6 Supplementary Material). Thus, there was partial support for the hypothesis that self-compassion and interpersonal problems mediate the relationship between insecure attachment and emotional distress.

When all of the above mediations were repeated as single mediation models, with self-compassion and interpersonal problems entered separately, the results did not notably change. This supports the findings that attachment avoidance is linked to emotional distress indirectly through low self-compassion, but not interpersonal problems.

The results of the regression analysis when depression was included as the DV indicated that the overall model was not significant (*F*(3, 65) = 2.59, *p* = 0.06, ns). Mediation analysis indicated that there was no significant overall indirect effect (*b* = 0.35, 95% BC CIs [−0.07, 0.95]).

## Discussion

This study investigated if self-compassion mediates the relationship between attachment and psychopathology in a clinical population. Our results suggest that low self-compassion, attachment insecurity and greater interpersonal problems are associated with greater levels of emotional distress in patients with mixed anxiety and depression. Specifically, lower levels of self-compassion and higher attachment-related avoidance individually predicted more anxiety and overall emotional distress. Greater levels of interpersonal problems were associated with, but did not significantly predict, anxiety, depression or overall emotional distress. The current study also illustrated that lower self-compassion and more interpersonal problems were associated with greater levels of attachment-related insecurity in clinical patients, replicating findings from non-clinical samples (e.g. Haggerty et al. 2009; Raque-Bogdan et al. 2011).

The main mediation model tested the relationships between attachment-related avoidance, interpersonal problems, self-compassion and overall emotional distress. Preacher and Hayes (2008) stated that it is possible to have significant indirect effects, even when there is no significant total indirect effect, and that this is common in multiple mediator models. Indeed, whilst we have not observed a significant overall indirect effect, there was an indirect pathway of attachment-related avoidance on emotional distress through self-compassion. This is in line with findings from non-clinical populations (Raque-Bogdan et al. 2011) and suggests that one reason individuals with higher levels of attachment-related avoidance experience emotional distress is through being unable to be compassionate towards the self. Contrary to our hypothesis however, interpersonal problems did not mediate the relationship between attachment and emotional distress. These results are consistent with the theoretical prediction that the development of self-compassion is rooted in early attachment experiences, with negative attachment experiences resulting in reduced capacity to be compassionate towards the self (Gilbert 2010).

The subsequent mediation analysis exploring anxiety and depression as separate constructs provided additional support for the hypothesis that self-compassion mediates the relationship between attachment and emotional distress. Both self-compassion and interpersonal problems were found to mediate the relationship between attachment-related avoidance and anxiety, and the total indirect effect was also significant. This remained the case when depression was controlled for, although only the pathway through self-compassion remained significant in this analysis. This is perhaps not surprising given self-compassion correlated with anxiety, but not depression, whilst interpersonal problems correlated with both constructs, suggesting that in this sample interpersonal problems had a stronger relationship with depression than self-compassion did. No significant findings were present when the mediation model with depression was tested, which could be due to the lack of relationship between attachment avoidance and depression.

It has been proposed that the relationship between attachment anxiety and self-compassion may be more straightforward than the relationship between attachment avoidance and self-compassion, due to those with attachment anxiety have a negative internal working model, whilst those with attachment avoidance potentially have a positive internal working model (Wei et al. 2011). Previous studies have consistently reported an association between higher levels of attachment anxiety and lower levels of self-compassion, whilst the relationship between attachment avoidance and self-compassion has produced mixed findings (Neff and McGehee 2010; Raque-Bogdan et al. 2011; Wei et al. 2011). In the current study, there did not appear to be any difference in the strength of the relationship between both styles of insecure attachment and self-compassion. Thus, this suggests that even though individuals with attachment avoidance may have a positive self-image, they may still struggle to self-soothe as a result of a lack of comfort from early attachment figures (Gilbert 2005; Gillath et al. 2005).

This study also has yielded unexpected findings. Specifically, attachment-related anxiety was not significantly associated with overall emotional distress, anxiety or depression. This may be related to the use of the ECR-R to measure attachment, which does not measure attachment security. Therefore, an individual with low levels of attachment-related anxiety may be securely attached and produce less polarised results compared to those in studies that classify attachment, including attachment security. Furthermore, neither attachment-related avoidance nor self-compassion was significantly associated with depression. Whilst these findings appeared to be contrary to those of previous research (MacBeth and Gumley 2012; Mikulincer and Shaver 2010), it is worth noting that the depression score was relatively low in this sample (just within the mild range) and was significantly lower than the anxiety score. Thus, it could be that the current sample was skewed towards including a greater number of participants with anxiety, and therefore underpowered for detecting a significant correlation in those with more severe symptoms of depression. In addition, despite the HADS being a widely used measure, previous literature indicates that the two subscales do not always assess independent symptoms of anxiety and depression, with strong correlations between them often indicated (Cosco et al. 2012). In the current study, although multicollinearity was not indicated, there was a significant correlation between the two subscales of the HADS. As such, it may be that this measure was not sensitive enough to detect independent symptoms of anxiety and depression.

Although in the predicted direction, the hypothesised relationship between self-compassion and interpersonal problems was not statistically detected. This relationship has not previously been explored and thus no comparisons to previous findings can be made. Based on Neff’s (2003) conceptualisation of self-compassion, it was predicted that more interpersonal problems would be associated with lower levels of self-compassion. Interestingly, Baker and McNulty (2011) hypothesised that higher levels of self-compassion could potentially lead to more interpersonal problems and less satisfaction with relationships. This study argued that those with high levels of self-compassion should have greater self-esteem, and therefore may feel less motivated to correct interpersonal mistakes due to feeling they should be accepted despite their flaws. On the other hand, less self-compassionate individuals may experience lower self-esteem and therefore may feel more motivated to deal with interpersonal mistakes to build social acceptance. Furthermore, Baker and McNulty (2011) found that the implications of self-compassion differed for men and women: for women, self-compassion was positively associated with motivation to resolve interpersonal mistakes and relationship problems. However, for men, this relationship was moderated by the level of conscientiousness. No gender differences were found in the current sample. Nevertheless, the above studies suggested that the relationship between self-compassion and interpersonal problems may be mediated or moderated by many factors. Future research is required to unpack this complexity further.

Finally, the hypothesis that those with attachment-related avoidance will experience emotional distress because of increased interpersonal problems was not supported. This differs from previous research in non-clinical populations where a significant mediation effect of interpersonal problems has been found (Hankin et al. 2005). As already noted, it may be that interpersonal problems are more strongly related to depression, which future research could examine by recruiting a population of participants diagnosed with Major Depressive Disorder. Another possible explanation is that ‘interpersonal problems’ is a broad term covering a range of difficulties, highlighted by the fact the IIP-32 consists of eight subscales. It may therefore be that different types of interpersonal problems have differing relationships with both attachment and mental health. In this study, we did not look into the effects on individual subscales due to a limited sample size. Future research is warranted to assess the hypothesised relationship between attachment, interpersonal problems and mental health in more detail.

### Limitations

Firstly, this study included a sample of participants who presented with mixed anxiety and depressive symptoms. As noted previously, the HADS does not necessarily assess anxiety and depression as separate constructs (Cosco et al. 2012). Therefore, the results of our additional mediation analysis treating anxiety and depression as separate outcomes should be interpreted with caution. On reflection, it would also be helpful to record the clinical diagnosis of the participants. Secondly, as this study was cross-sectional in design, conclusions regarding causation cannot be drawn. Thus, whilst self-compassion was found to be a significant mediator, it is possible that alternative models may exist that provide a good fit to the data. For example, it may be that self-compassion is dependent on mood state, rather than being a causal trait-like factor for emotional distress. As mentioned in “Introduction”, the link between interpersonal problems and psychopathology is likely to be bidirectional; as such, interpersonal problems could also be hypothesised as an indicator of psychopathology and be examined as an outcome rather than a mediator. Thus, longitudinal studies are required to clarify the direction of causation.

The current study also relied on self-report measures. Although the questionnaires used were deemed valid for the population being assessed, they do have limitations. For example, the psychometric properties of the SCS have been questioned due to the inability to replicate the six-factor structure in non-student populations (Costa et al. 2015; López et al. 2015; Williams et al. 2014). It is noteworthy that the Cronbach’s alpha for the SCS in the current study was lower than has been reported in previous studies. This difference could be due to the current study involving a clinical sample, whereas the original psychometric properties of the scale were established in non-clinical samples. In addition, the SCS involves three positive (self-kindness, common humanity and mindfulness) and three negative (self-judgement, isolation and overidentification) subscales. Recently, there has been a debate regarding the factor structure of SCS; whilst Neff (2016) argued in favour of the use of total score and the original six subscales, it has been argued that the positive and negative items are measuring different aspects of self-compassion and therefore should not be combined to provide a total self-compassion score (López et al. 2015). A recent meta-analysis of 18 studies has further suggested that whilst the positive and negative items are associated with psychopathology in the expected direction, negative items are stronger predictors of psychopathology (Muris and Petrocchi 2017). As our current sample size has just reached our minimum target, it was not sufficiently powered to support secondary analyses based on these subscores. Future research may consider assessing self-compassion in alternative ways and/or analysing SCS based on these two subscales in a larger sample.

## Electronic Supplementary Material

ESM 1 (DOCX 17 kb)
