# Supplementary material for: The Relationships Between Self-Compassion, Attachment and Interpersonal Problems in Clinical Patients with Mixed Anxiety and Depression and Emotional Distress
Source: Mindfulness (N Y). 2017 Nov 6;9(3):961–71. doi: 10.1007/s12671-017-0835-6 (PMC5968043; doi:10.1007/s12671-017-0835-6)
Supplement: Supplementary file 1 — (DOCX 17 kb) [file 12671_2017_835_MOESM1_ESM.docx]

Table 5: Bootstrapped indirect effects of potential mediators: emotional distress (supplementary materials)

| **Mediator** | **Point estimate** | **SE** | **Bootstrapping 95% BC CIs** | |
| --- | --- | --- | --- | --- |
|  | | | Lower | Upper |
| Self-compassion | 0.3217 | 0.2090 | **0.0219** | **0.8802** |
| Interpersonal problems | 0.3265 | 0.3309 | -0.2146 | 1.1196 |
| Total indirect effect | 0.6482 | 0.4117 | -0.0209 | 1.6395 |

*Note.* Bold denotes significant indirect effects

Table 6: Bootstrapped indirect effects of mediators: anxiety (supplementary materials)

| **Mediator** | **Point estimate** | **SE** | **Bootstrapping 95% BC CIs** | |
| --- | --- | --- | --- | --- |
|  | | | Lower | Upper |
| Self-compassion | 0.2081 | 0.1160 | **0.0301** | **0.4967** |
| Interpersonal problems | 0.2382 | 0.1401 | **0.0019** | **0.5763** |
| Total indirect effect | 0.4463 | 0.1831 | **0.1400** | **0.8780** |
| **Controlling for depression** | | | | |
| Self-compassion | 0.2081 | 0.1160 | **0.0369** | **0.4435** |
| Interpersonal problems | 0.2382 | 0.1401 | -0.0916 | 0.3910 |
| Total indirect effect | 0.4463 | 0.1831 | **0.0614** | **0.7008** |

*Note.* Bold type denotes significant indirect effects
